# Community perceptions of pre-eclampsia and eclampsia in southern Mozambique

**DOI:** 10.1186/s12978-016-0135-y

**Published:** 2016-06-08

**Authors:** Helena Boene, Marianne Vidler, Charfudin Sacoor, Abel Nhama, Ariel Nhacolo, Cassimo Bique, Pedro Alonso, Diane Sawchuck, Rahat Qureshi, Eusébio Macete, Clara Menéndez, Peter von Dadelszen, Esperança Sevene, Khátia Munguambe

**Affiliations:** Centro de Investigação em Saúde da Manhiça (CISM), Manhiça, Mozambique; Department of Obstetrics and Gynaecology, and the Child and Family Research Unit, University of British Columbia, Vancouver, British Columbia Canada; Ministério da Saúde, Maputo, Mozambique; Hospital Central de Maputo, Maputo, Mozambique; Barcelona Institute for Global Health (ISGlobal)/Hospital Clinic - Universitat de Barcelona, Barcelona, Spain; Division of Women and Child Health, Aga Khan University, Karachi, Sindh Pakistan; Universidade Eduardo Mondlane, Faculdade de Medicina, Maputo, Mozambique

**Keywords:** Africa South of the Sahara, Eclampsia, Hypertension, Maternal mortality, Attitudes, Mozambique, Pre-eclampsia, Seizures, África subsaariana, Eclâmpsia, Hipertensão, Mortalidade materna, Atitudes, Moçambique, Pré-eclâmpsia, Convulsões

## Abstract

**Background:**

Sub-Saharan Africa has the highest maternal mortality ratio at 500 deaths per 100,000 live births. In Mozambique maternal mortality is estimated at 249-480 per 100,000 live births and eclampsia is the third leading cause of death. The objective of this study was to describe the community understanding of pre-eclampsia and eclampsia, as a crucial step to improve maternal and perinatal health in southern Mozambique.

**Methods:**

This qualitative study was conducted in Maputo and Gaza Provinces of southern Mozambique. Twenty focus groups were convened with pregnant women, partners and husbands, matrons and traditional birth attendants, and mothers and mothers-in-law. In addition, ten interviews were conducted with traditional healers, matrons, and a traditional birth attendant. All discussions were audio-recorded, translated from local language (*Changana*) to Portuguese and transcribed verbatim prior to analysis with QSR NVivo 10. A thematic analysis approach was taken.

**Results:**

The conditions of “pre-eclampsia” and “eclampsia” were not known in these communities; however, participants were familiar with hypertension and seizures in pregnancy. Terms linked with the biomedical concept of pre-eclampsia were *high blood pressure, fainting disease* and *illness of the heart*, whereas *illness of the moon*, *snake illness*, *falling disease*, *childhood illness*, *illness of scares*and *epilepsy* were used to characterizeeclampsia. The causes of hypertension in pregnancy were thought to include mistreatment by in-laws, marital problems, and excessive worrying. Seizures in pregnancy were believed to be caused by a snake living inside the woman’s body. Warning signs thought to be common to both conditions were headache, chest pain, weakness, dizziness, fainting, sweating, and swollen feet.

**Conclusion:**

Local beliefs in southern Mozambique, regarding the causes, presentation, outcomes and treatment of pre-eclampsia and eclampsia were not aligned with the biomedical perspective. The community was often unaware of the link between hypertension and seizures in pregnancy. The numerous widespread myths and misconceptions concerning pre-eclampsia and eclampsiamay induceinappropriatetreatment-seeking and demonstrate a need for increased community education regarding pregnancy and associated complications.

**Trial Registration:**

NCT01911494

**Electronic supplementary material:**

The online version of this article (doi:10.1186/s12978-016-0135-y) contains supplementary material, which is available to authorized users.

## Resumo

**Antecedentes:**

A África Subsaariana tem a taxa de mortalidade materna mais alta, com cerca de 500 mortes por 100.000 nascidos vivos. Em Moçambique a mortalidade materna é estimada em 249-480 por 100.000 nascidos vivos e a eclâmpsia é a terceira causa de morte. O objectivo deste estudo foi de descrever o que as comunidades entendem por pré-eclâmpsia e eclâmpsia, como uma etapa crítica para melhorar a saúde materna e perinatal no sul de Moçambique.

**Métodos:**

Este estudo qualitativo foi realizado nas Províncias de Maputo e Gaza no sul de Moçambique. Vinte discussões em grupos focais foram organizadas com mulheres grávidas, parceiros e maridos, matronas e parteiras tradicionais, mães e sogras. Adicionalmente, dez entrevistas em profundidade foram feitas com praticantes de medicina tradicional, matronas e parteiras tradicionais. Todas as entrevistas e discussões foram áudio-gravadas, traduzidas da língua local (*Changana*) para Português e transcritas na íntegra antes da análise com QSR NVivo 10. Foi usada uma abordagem de análise temática.

**Resultados:**

As condições de “*pré-eclâmpsia*” e “*eclâmpsia*” não eram conhecidas nestas comunidades; contudo, os participantes estavam familiarizados com hipertensão e convulsões na gravidez. Os termos ligados a conceitos biomédicos de pré-eclâmpsia eram tensão alta, desmaios e doença do coração; enquanto doença da lua, doença da cobra, doença de queda, doença de infância, doença de sustos e epilepsia eram os termos usados para caracterizar a eclâmpsia.

Localmente, pensa-se que as causas de hipertensão na gravidez incluem maus tratos por parte dos sogros, problemas conjugais e excesso de preocupações. Em relação as convulsões na gravidez, acredita-se que sejam causadas por uma cobra que vive dentro do corpo da mulher. Os sinais de perigo que se pensa serem comuns as duas condições eram dor de cabeça, dor do peito, fraqueza, tonturas, desmaios, sudação e pernas inchadas.

**Conclusão:**

Crenças locais no sul de Moçambique em relação as causas, apresentação, resultados e tratamento da pré-eclâmpsia e eclâmpsia não estavam alinhadas com a perspectiva biomédica. A comunidade muitas vezes não tinha conhecimento da ligação entre a hipertensão e as convulsões na gravidez. Os numerosos e generalizados mitos e falsos conceitos relativos a pré-eclâmpsia e eclâmpsia podem levar a tratamento inapropriado – exigindo e demonstrando a necessidade de reforço na educação da comunidade em relação a gravidez e complicações associadas.

## Background

Improving maternal health is one of the Millennium Development Goals (MDGs) adopted by the international community in 2000. According to MDG5, countries committed to reduce global maternal deaths by three quarters by 2015 [1]. Despite the significant gains in terms of reduction, in 2015, an estimated 303,000 maternal deaths will occur globally, representing a decline of only 43 % since 1990 (estimated 535,000 maternal deaths) and a similar reduction since the adoption of the MDGs in 2000 (estimated 529,000 deaths) [2], which is far from the target 75 % reduction.

Sub-Saharan Africa has the highest maternal mortality ratio (MMR), with 546 deaths per 100,000 live births in 2015 [2]. In Mozambique, maternal-newborn health is a public health priority; as it is reflected in recent government investments and policies. The implementation of the National Strategic Plan for the Reduction of Maternal and New Born Mortality since 2000, has led to improvements in access to quality health services including antenatal care, family planning, and the diagnosis and treatment of obstetric complications [3]. The MMR in Mozambique has decreased from 1,300 in 1990 [4] to current estimates of 249–480 per 100,000 live births [5]. The major direct causes of maternal mortality in Mozambique are obstetric, such as postpartum haemorrhage, rupture of the uterus, and puerperal sepsis, with an increasing role of infectious diseases mainly HIV/AIDS, tuberculosis and malaria [3, 6]. Eclampsia has been reported to be the third leading cause of maternal death in the country [3, 7]. Effective management of pre-eclampsia and eclampsia is limited to hospital-based treatment, thus increasing women’s probability for severe complications or death due to delays in reaching emergency care [8].

A previous study conducted in Mozambique concluded that 13 % of women with eclampsia reported not having had blood pressure monitoring during antenatal care (ANC) [9]. This finding highlights the need for improvements in case detection at the primary care level, increased community awareness of the danger signs of eclampsia, as well as prompt referrals. Community-level detection and basic management of pre-eclampsia and eclampsia are needed to effectively address these gaps.

An understanding of community perspectives is crucial to the successful implementation of any community-based intervention. Literature regarding community perceptions of the hypertensive disorders of pregnancy is scarce, particularly in Africa. In a study conducted in Malawi [10] few women were able to correctly identify pre-eclampsia or eclampsia as maternal health problems. Similarly, studies in Nigeria showed limited knowledge of these complications and many misconceptions regarding the causes, as well as regular use of potentially harmful traditional practices [11, 12]. In a study on community perceptions of maternal morbidity in Mozambique, pregnant women identified malaria, abdominal, back and body pains as the most important ailments during pregnancy, neither pre-eclampsia nor eclampsia were mentioned [13]. There are no previous studies describing community beliefs and practices for pre-eclampsia or eclampsia in Mozambique.

The focus of this article is to document community knowledge, attitudes and beliefs regarding pre-eclampsia and eclampsia in southern Mozambique. A better understanding of community perspectives could be useful for policy makers and health care providers to implement effective strategies to improve maternal health care delivery. These results are equally important to guide future community-based interventions, and to assess the effectiveness of innovative packages of care to control and mitigate pre-eclampsia and eclampsia.

## Methods

### Study area

**Fig. 1:**
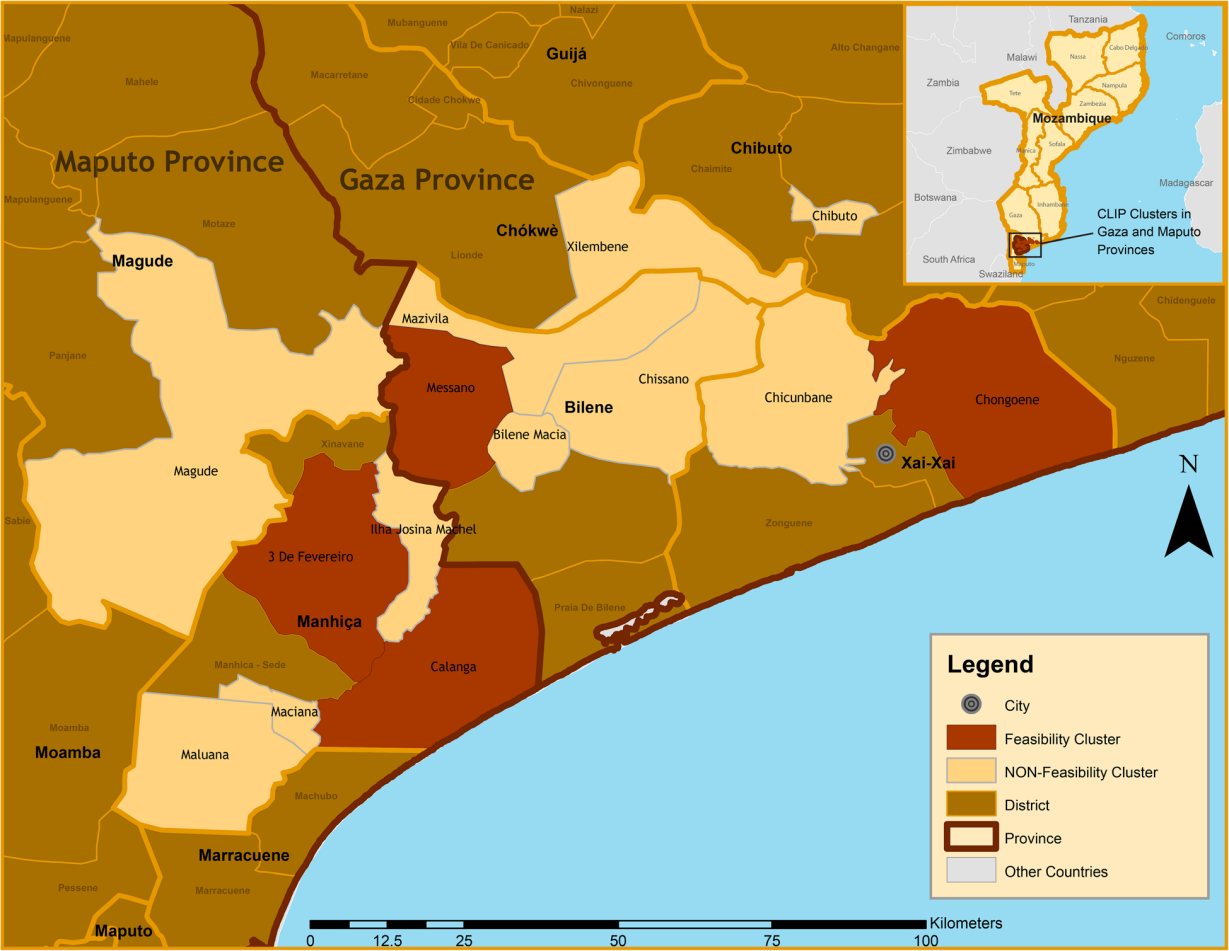
Map of study areas, southern Mozambique

This is an ancillary study of a multinational cluster randomized control trial in Nigeria, Mozambique, Pakistan and India (the Community Level Interventions for Pre-eclampsia trial-CLIP) (NCT01911494) [14]. For this qualitative study, four study regions in Mozambique were selected, two from Maputo Province and two from Gaza Province (Fig. 1). Each study region was equivalent to an Administrative Post (AP), with the exception of Ilha Josina Machel and Calanga administrative posts, which were combined for the purposes of fulfilling the minimal population size for a study cluster within the context of the CLIP trial, and given that they are neighbouring APs. Each region was purposively selected to reflect a variety of socioeconomic and demographic characteristics, such as level of urbanization, population density, distance to a trading centre, and presence of a referral facility.

The Ilha Josina Machel-Calanga region is located in north-east Maputo Province, populated mainly by farmers and fishermen. This area is characterised by extremely poor transportation networks, which further deteriorate due to flooding in the rainy season. Três de Fevereiro is located in the north of Maputo Province, it is intersected by the 1^st^ National Road (the major two-way highway in Mozambique, and the only connection between the northern, central and southern regions of the country) and has reasonable infrastructure such as modern communication networks, some secondary roads, and public services. Most residents of this AP are employed by the Xinavane Sugar Company and other private sugar and rice farms. This area is an important informal business centre, with a large sector of the young adult male population employed in the mining sector in South Africa.

The two regions in Gaza Province were Messano and Chongoene. Messano,in the southwest, has a weak community infrastructure set-up including poor access to the main road. The primary occupation of residents is small-scale farming. Chongoene is a coastal region in northern Gaza. It is the newly appointed district head office, which has led to improvements in commerce, administrative services, tourism, and the agriculture sector.

Most residents of the four regions belong to the Changana ethnic group. The predominant occupation is farming, especially among women. Raising livestock, informal trading, and handicrafts are the other sources of income. Most men migrate to South Africa, Swaziland and other cities in Mozambique for work. Education indicators vary between the two provinces, with a 22 % illiteracy rate in Maputo and 38 % in Gaza, in both cases literacy is lowest among women [14]. For more detailed study site characteristics see Table 1.

**Table 1:** Study site characteristics

Characteristics	Study Regions				
	Ilha Josina Machel	Calanga	Três de Fevereiro	Messano	Chongoene
Population	8591	14,988	46,388	13,400	32,760
Population of women of reproductive age (12–49)	1706	2074	11,694	4825	11,276
Number of villages	2	2	3	3	6
Number of health facilities	1	1	4	2	6
Predominant language	Changana	Changana	Changana	Changana	Changana
Predominant religion	Zion	Zion	Zion	Zion	Zion

Source: Unpublished data from demographic census(2014) and demographic rounds (2015) of the CLIP study

### Data collection

This article is a component of a larger formative study prior to the CLIP trial. While the formative research was based on a mixed methods approach, the present article focuses on the qualitative component, comprised of focus group discussions and in-depth interviews with community stakeholder groups (see Tables 2 and 3 for participant characteristics).

**Table 2:** Characteristics of focus group discussion participants

	Stakeholder group	Region	# of participants	Age (median)	Marital status	Occupation	Schooling level
1	Women of reproductive age	Ilha Josina Machel﻿^a^	9	30	Married (9)	Farmer (9)	Never studied (3) Primary (6)
2		Calanga^a^	3	25	Married (3)	Farmer (3)	Primary (3)
3		Três de Fevereiro	8	23	Married (8)	Farmer (8)	Never studied (1) Primary (4) Secondary (3)
4		Messano	8	28	Married (6) Widow (1) Single (1)	Farmer (7) Nurse (1)	Primary (7) Secondary (1)
5		Chongoene	*Unknown* ^*b*^	*Unknown*	*Unknown*	*Unknown*	*Unknown*
6	Mothers and mothers-in-law	Ilha Josina Machel﻿	12	29	Married (7) Widow (5)	Farmer (12)	Never studied (4) Primary (8)
7		Calanga	12	59	Married (7) Widow (3) Divorced (2)	Farmer (12)	Never studied (1) Primary (11)
8		Três de Fevereiro	*Unknown*	*Unknown*	*Unknown*	*Unknown*	*Unknown*
9		Messano	8	39	Married (5) Widow (1) Single (2)	Farmer (8)	Never studied (6) Primary (2)
10		Chongoene	11	46	Married (3) Widow (1) Single (7)	Housewife (11)	Never studied (3) Primary (8)
11	Partners and husbands	Ilha Josina Machel﻿	12	37	Married (12)	Farmer (12)	Never studied (3) Primary (8) Secondary (1)
12		Calanga	4		Married (4)	Farmer (1) Fisherman (1) Traditional healer (1) Locality chief (1)	Never studied (1) Primary (3)
13		Três de Fevereiro	10	45	Married (10)	Farmer (2) Security (2) Seller (1) Mason (1) Small jobs (2) Gas station clerck (1)	Primary (10)
14		Messano	6	42	Married (6)	Farmer (6)	Primary (6)
15		Chongoene	7	49	Married (3) Single (4)	Farmer 6) Driver (1)	Primary (7)
16	Matrons and traditional birth attendants	Ilha Josina Machel﻿	6	55	Married (3) Widow (3)	Farmer (6)	Never studied (5) Primary (1)
17		Calanga	9	67	Married (3) Widow (4) Divorced (2)	Farmer (9)	Primary (9)
18		Três de Fevereiro	12	65	Married (4) Widow (7) Divorced (1)	Farmer (12)	Never studied (9) Primary (3)
19		Messano	10	43	Married (5) Widow (3) Single (2)	Farmer (8) Teacher (1) Housewife (1)	Never studied (1) Primary (7) Secondary (2)
20		Chongoene	9	58	Married (2) Widow (1) Single (6)	Housewife (9)	Never studied (7) Primary (2)

^a^Despite the fact that these two Administrative Posts were combined into one single cluster, the data was collected separately

^b^Missing data

**Table 3:** Characteristics of interview participants

	Stakeholder group	Region	Age	Gender	Marital status	Schooling level
1	Traditional healers	Ilha Josina Machel﻿	61	Male	Married	Primary
2		Calanga	35	Male	Married	Primary
3		Três de Fevereiro	44	Female	Widow	Primary
4		Messano	35	Female	Widow	Primary
5		Chongoene	49	Female	Married	Primary
6	Matrons	Ilha Josina Machel﻿	89	Female	Widow	Primary
7		Calanga	78	Female	Married	Primary
8		Três de Fevereiro	81	Female	Married	Primary
9		Messano	65	Female	Married	Primary
10		Chongoene	49	Female	Single	Primary

Focus groups were chosen to best capture community members’ views, while enabling open discussion between participants. It was difficult to convene focus groups for traditional healers and matrons due to the limited number available; therefore individual interviews were conducted with these two stakeholder groups.

Data collection took place between September 2013 and May 2014. This process was conducted by a team comprising a Mozambican social scientist and four trained interviewers, all employed by the Manhiça Health Research Centre (CISM). All data collectors were fluent in Portuguese and Changana, the predominant local language.

As part of the rapport-building stage, the first contact was made with the community chief at the Administrative Post level, to obtain permission for data collection. Following this, a neighbourhood was randomly selected for data collection within each AP. Neighbourhood chiefs (known as *secretários dos bairros)* supported the study team in the identification of participants who fulfilled the inclusion criteria for interviewsand focus groups. Participants had to belong to one of the following categories:pregnant, partners or husbands of women of reproductive age (WRA), mothers or mothers-in-law of WRA, matrons or traditional birth attendants (TBA), elders and traditional healers. The team made the final selection by verifying the characteristics of the potential participantsand the number needed for interviews and focus groups. The *secretários dos bairros* were instructed to identify participants from different *quarteirões* (the set of houses located in the same block within a *bairro*).

Focus groups were conducted either at the *círculos* (the usual community gathering location), or at the community leaders’ house, as groups could easily be convened in these locations. A total of 20 focus groups were conducted with an average of 7 [6–14] participants in each session. Groups were homogeneous according to the main inclusion criterion. However, there was heterogeneity within each focus group in terms of age, residence (*quarteirão*), occupation and education, as captured in Table 2. Each discussion lasted for 30 to 80 min.

A total of 10 interviews were conducted with community members (traditional healers and matrons). Interviews were conducted one-on-one in the home or workplace of participants, and were 30–60 min in length.

Data collection instruments served asguides for the discussions, allowing for probing and follow-up questions whenever necessary. These interview and focus group guides had been usedin Nigeria, India and Pakistan in the context of the CLIP trial, and were subsequently adapted to the local context during the piloting process in Mozambique. Theguides differed slightly according to the stakeholder groups, but in general they touched upon similar themes.

Although the guides were written in Portuguese, data collection was conducted primarily in the Changanalocal language. The choice of language was determined by participants’ preference.

Ethical approval for this study was granted by the CISM Institutional Review Board (CIBS_CISM/08/2013), as well as by the University of British Columbia in Canada (H12-00132).

### Data management and analysis

Focus group discussions (FGD) and in-depthinterviews (IDI) were digitally recorded using Olympus AS-2400 PC®; IDIs and FGDs were transcribed verbatim and translated simultaneously from Changana to Portuguese for analysis at CISM. On site, quality control was ensured by a secondary review of 20 % of the transcripts against the audio recordings to confirm accuracy. Two social science researchers coded all the data, which was originally transcribed in Portuguese, in Mozambique. Twenty-six percent of all transcripts were translated into English and re-analysed by an external collaborator from UBC for quality controlandto contribute to interpretation of the data. Data from Ilha Josina Machel and Calanga were analysed separately and subsequently combined for presentation of qualitative findings.

**Fig. 2:**
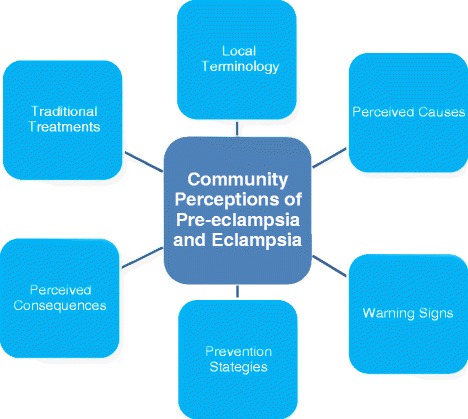
Thematic categories used in analysis

Data saturation was sufficiently met after 20 focus group discussions and 10 individual interviews. Data analysis was performed using NVivo version 10.0 (QSR International Pty.Ltd. 2012). A thematic analysis approach was taken. The coding structure was developed in advance of analysis through collaboration among researchers. Themes were subsequently adjusted and new themes were added as they emerged from the data (Fig. 2).

## Results

### Participants’ characteristics

Focus group participants were between 18 and 87 years of age. More than half (55 %) had completed primary education and the majority were farmers. Sixty five percent of the participants were married (Table 2). Interview participants were male (30 %) and female (70 %) between 35 and 79 years old, most had completed primary education (80 %), and over half (60 %) were married (Table 3).

### Local terminology

The terms “pre-eclampsia” and “eclampsia” were not known to study participants. However, when facilitators further described the presentation of these conditions, participants did recognize them, yet they used alternative local terms. Specifically, pre-eclampsiawasreferred to as *tensão alta* (high blood pressure) or simply *tensão* (pressure). These were the only terms which were mentioned in Portuguese. The other terms used were in the local dialect, Changana: *mavabji ya mbilo* or *mavabji ya timpfalu* (illness of the heart) and *mavabji ya kuwa* (fainting disease). The Portuguese term used for eclampsia was *epilepsia* (epilepsy) and in Changanaeclampsia was referred to as *mavabji ya nweti* (illness of the moon), *nhocane* (little snake), *mavabji ya mbilo* (illness of the heart), *mavabjiyakuwa*(falling disease), *mavabji ya vatsonguana* (children’s illness), *mavabji ya makulo* (the big illness) and *mavabji ya kudzuka* (illness of scares) (Tables 4 and 5).

**Table 4:** Local names and perceived causes of pre-eclampsia

Pre-eclampsia	
Local names	High blood pressure (*tensão alta*)^a^ Pressure (*tensão*)^a^ Illness of the heart (*mavabjiyambilo* or *mavabjiyatimpfalu*)^b^ Fainting disease (*doença de desmaiar*)^a^
Perceived causes	Mistreatment by the in-laws Marital problems Excessive thinking/worrying Anger Sadness Eating a diet high in salt

^a^Terms mentioned in Portuguese

^b^Term mentioned in Changana

**Table 5:** Local names and perceived causes of eclampsia

Eclampsia	
Local names	Illness of the moon (*mavabjiyanweti*)^b^ Snake illness (*nhocane*)^b^ Illness of the heart (*mavadjiyambilo*)^b^ Falling disease (*doença de cair*)^*a*^ Children’s illness (*mavabjiyavatsonguana*)^b^ Big illness (*mavabjiyamakulo*)^b^ Illness of scares (*mavabjiyakudzuka*)^b^ Epilepsy (*epilepsia*)^*a*^
Perceived causes	Snake (*nhocane*)^b^

^a^Terms mentioned in Portuguese

^b^Terms mentioned in Changana

### Perceived causes

Neither pre-eclampsia nor eclampsia was perceived to be conditions specific to pregnancy. In addition, community participants rarely described a relationship between thetwo conditions. In these discussions with communities there was limited knowledge of the origin of pre-eclampsia. They most often related pre-eclampsia to marital problems, such as mistreatment by in-laws, strenuous work, excessive thinking or worry, anger, and sadness.

*“You will get sick if you think a lot, thinking excessively about something and if you talk to yourself inside your heart and if something doesn’t go well in your head, all this can lead to that disease.”* Mother or mother-in-law, Ilha Josina Machel-Calanga

It was not common to associate pre-eclampsia with lifestyle factors; however, a few participants did believe that diets rich in salt may contribute to the occurrence of pre-eclampsia. During focus groups, all matrons claimed pre-eclampsia was caused by modern ways of living, as the condition was thought to be rare in previous generations (Table 4).

Although there were widespread cultural beliefs surrounding convulsions, these were not specific to pregnancy. Seizures of all types were associated with a childhood illness, known as the *mavabji ya nweti* (illness of the moon). This illness is thought to be caused by a small snake (*nhocane*) which lives in the abdomen; it is believed that the phases of the moon can create imbalances in the body, which include growth abnormalities, digestive problems, and ultimately seizures. The ‘illness of the moon’ is believedto be congenital and continues without appropriate treatment in childhood. Those who are not treated maysuffer in pregnancy, which may affectbirth outcomes. Furthermore, seizures are believed to be contagious to children, therefore they are usually sent away when someone is seizing (Table 5).

*“Hum what must be done if you find somebody in this way? In a house where there are children or other people […] this person must be carried away and placed somewhere, and afterwards you must find somebody nearby who knows the rules, then they will be able to help her.”* Partner or husband, Ilha Josina Machel-Calanga

### Warning signs

Community participants described the warning signs for pre-eclampsia as headache, heart pain, a strong heart-beat, burning sensation in the chest, shortness of breath, loss of speech, weakness, dizziness, fainting, sweating and swollen feet. Many of the warning signs for eclampsia were common with those mentioned for pre-eclampsia. Those warning signs unique to seizures in pregnancy included falling, eyes rolling back and red eyes.

*“She always feels pain in the heart, or feels her body is weak. When you do hard work while you suffer from this disease, the heart may throb, you may even have dizziness and then faint.”* Woman of reproductive age, Ilha Josina Machel-Calanga

### Prevention strategies

The majority of participants expressed that early ANC and regular visits to the health facility are the most effective measures to prevent pregnancy complications.

*“Pregnant women have to go to the hospital while the pregnancy is still small, growing pregnancy while going to the hospital.”* Mother or mother in-law, Três de Fevereiro

The only community-level prevention of seizures discussed wasthe administration of a medicine that consisted of a mixture of roots prepared in a clay pot *(swimbitana),* which must be taken daily during childhood to be effective later in life. In addition, there are practices in pregnancy not specific to pre-eclampsia or eclampsia that are followed in order to ensure well-being, such as: avoiding bitter foods and drinks, avoiding strenuous labor, and small lacerations on the breast for the application of traditional powders.

### Perceived consequences

The possible outcomes of pre-eclampsia, according to the community were miscarriage, premature delivery and death. Seizures in pregnancy were seen to be dangerous to women and infants because they may cause serious injury, paralysis, or death. These pregnancy complications are thought to be most dangerous before delivery, when pregnant women are more vulnerable, as was mentioned below during a focus group discussion.

*“Usually it is dangerous during pregnancy, but after birth some things decrease. It is not as if you were pregnant like that woman, and she is ill she will have the same strength as a non-pregnant woman, then one woman that is like that can go wrong”* Partner or husband, Ilha Josina Machel-Calanga

Paralysis of limbs due to falling from a seizure was perceived to be an immediate consequence of eclampsia. In addition, participants described that both mother and infant are vulnerable to death. It was believed that the probability of death is increased as a result of inappropriate behaviours of those surrounding the woman, for instance expressing negative emotions.

*“Another thing is that when she falls nobody must cry, because if she falls down and does not die but someone cries, she loses strength and dies. The people must be calm and strong.”* Partner or husband, Três de Fevereiro

### Traditional treatments

Most community members interviewed believed there is no traditional cure for pre-eclampsia or eclampsia; it can only be treated traditionally in childhood. Despite this, if a pregnant woman is unconscious she can be revived by exposing her to strong odours such as dirty shoes or leaves from *mafurreira* (a local wild fruit tree), lemon tree, *maungua-unguana* (a wild plant) or tobacco. Women who suffered seizures in pregnancy were usually taken to the shade or a place that allows her to get fresh air.

## Discussion

To the knowledge of the authors, this is the first qualitative study comprehensively describing the local terminology, causes, consequences as well as local preventive and treatment practices for pre-eclampsia and eclampsia by communities in Mozambique. Studies of this nature have been conducted elsewhere, mostly in Malawi, Nigeria and Latin America, where participants were women who had experienced pre-eclampsia, or their close relatives [11, 15, 16]. It is, however, important to also understand perceptions of the wider community.

Pre-eclampsia and eclampsia are terms unknown to the communities involved in this study, which is different from findings from one of the above studies, where eclampsia was mentioned by Nigerian women as one of the main causes of maternal mortality [17]. Nevertheless, the present study revealed a basic awareness of hypertension and seizures in pregnancy. This finding supports the need to reinforce education regarding pregnancy complications.

Despite the awareness, among some groups, regarding some pre-eclampsia and eclampsia warning signs, they did not view pre-eclampsia or eclampsia as uniquely associated with pregnancy. This assertion is illustrated by the fact that they are often depicted by the combination of traditional terminologies which do not enclose any reference to “pregnancy” or “pregnant women”. Seizures are generally viewed as a childhood condition, caused by supernatural forces that can persist through adulthood if untreated. Similarly, a study conducted in Nigeria revealed that the origin of pre-eclampsia was related to supernatural causes. However,evil spirits, which were described as such supernatural elements in Nigeria, were not mentioned by participants of this study.

These misconceptions as well as the use of traditional treatments can lead to serious consequences,such as maternal or foetal death,resulting from inadequate or inappropriate treatment. The use of alternative treatment can also increasedelays in seeking care at the health facility, decreasing the chances of survival when help is eventually sought at the facility.

Other important perceived underlying factors for pre-eclampsia were social and emotional problems, often associated to gender roles within the household. Similar causes have been reported in Colombia where insufficient antenatal care, familial predisposition, and stress werethought to be responsible for pre-eclampsia [15]. It is concerning that in this setting, none of the perceived causes were pregnancy-related; moreover, there was no clear distinction of these conditions in pregnancy from similar conditions outside pregnancy, such as epilepsy or seizures associated to certainchildhood disorders. This finding suggests that pregnant women, and those who care for them in the community, may misunderstand the cause seizures and overlook the importance and heightened risk in pregnancy.

Notwithstanding, women were concerned about their vulnerability during pregnancy, as earlier reported in the same setting by Boene et al [13], and as evidenced by the range of warning signs and consequences reported in this study. Participants described similar warning signs for pre-eclampsia and eclampsia, which may further reflect limited familiarity with the conditions.

The serious outcomes discussed for pre-eclampsia and the community´s familiarity with possible consequences reflects a higher perceived severity of the condition in the community.

This study revealed a number of misconceptions rooted in cultural norms that ultimately shape the way people act and react, such as not crying when assisting woman with eclampsia and not approaching pregnant women with eclampsia in order to avoid becoming affected.

Participants did not report any traditional definitive cureor pre-eclampsia or eclampsia, apart from remedies that areused to alleviate seizures. On the contrary, traditional remedies, such as holy water, herbs, concoctions, and charms are commonly used and believed to be effectivefor these conditions in other African settings [11, 12].

The findings from this study, coupled with the positive tendency ofseeking antenatal care in this setting, highlight the opportunityto enhance community based health education in collaboration with maternal health care providers. This qualitative study contributes to the understanding of local beliefs around pre-eclampsia and eclampsiaand can support the identification of converging and contrasting views between the local and the biomedical perspectives. This improved understanding can inform the development of more appropriate health promotion messages for pregnant women, their family members and communities. This information can also be used to revisit training curricula for health professionals in the field of maternal health, in order to improve their awareness of sociocultural factors contributing to maternal morbidity and mortality in the regions they serve [18].

### Limitations of the study

As with any research there are a number of limitations to these findings. The data werecollected in four communities in Maputo and Gaza Provinces;although these results show good representation of the region, results are not generalizable to other settings. Due to translation of the data (between Changana, Portuguese and English), some subtleties of meanings may have been lost; however, strict quality control steps were put in place throughout the transcription,translation, and coding processes to minimize this limitation.

### Strengths of the study

Husbands and mothers-in-law are typically those with decision-making power in pregnancy; therefore, it is essential that these views have been explored and included in the findings. In addition, groups of differing ages and educational levels participated; this allowed findings to be applicable to a wider population. Interview facilitators were well trained, equipped with previous qualitative research experience, familiar with the community, and fluent in the local dialect. Relationships with the communities were established prior to data collection by approaching the administrative post chiefs for prior permission. This qualitative study included a substantial sample size with diversity in the types of participants and regions represented. Analysis was conducted as a team with the use of multiple researchers.

## Conclusions

In southern Mozambique, the terms pre-eclampsia and eclampsia are not known, but when prompted, the conditions are understood as hypertension and seizures, not necessarilyrelated to pregnancy. Local beliefs regarding the causes of these conditions are not aligned with the biomedical perspective, and instead are associated with supernatural, emotional and social causes, which may result ininappropriatecare-seeking decisions. Effective community-based interventions for the control of pre-eclampsia must be designed and implemented with the aim of increasing awareness of this condition, as well as the associated risks in pregnancy. Appropriate community-based and culturally sensitive educational materials should include information regarding causes, warning signs and consequences of pre-eclampsia and eclampsia. Health professionals serving these communities must be made aware of the local beliefs about pre-eclampsia and eclampsia.

### Key messages

1. In the southern Mozambicancommunities involved in this study, the concepts of pre-eclampsia and eclampsia were not fully understood. When further described, the terms for hypertension and convulsions were known but not associated with pregnancy.
2. Communities describedwarning signs of hypertension and convulsions during pregnancy, but these are associated to conditions outside pregnancy and not reflective of the biomedical model.
3. Communities do not provide traditional treatment for pre-eclampsia or eclampsia but rather perform specific actions to revive women with seizures.
4. Communities identifiedhealth facilities as the most appropriate place for management of pregnancy complications.
5. There is a need for interventions at the community-level to educate regarding pre-eclampsia and eclampsia: its causes, signs and symptoms, treatment and the need for referral.
6. There is need to make health professionals, especially those in training, aware of the local understanding of pre-eclampsia and eclampsia to ensure a more effective interaction between them and the communities they will serve.

### Peer review

Peer review reports for this article can be found in Additional file 1.

## Additional file

Additional file 1: Peer review reports. (PDF 151 kb)

## Abbreviations

ANC: antenatal care
AP: administrative post
CISM: Manhiça Health Research Centre
CLIP: community level interventions for pre-eclampsia
IDI: in-depth interview
MDG: millennium development goals
MMR: maternal mortality ratio
TBA: traditional birth attendants, FGD, focus group discussion
