# Supplementary material for: Community perceptions of pre-eclampsia and eclampsia in southern Mozambique
Source: Reprod Health. 2016 Jun 8;13(Suppl 1):33. doi: 10.1186/s12978-016-0135-y (PMC4943502; doi:10.1186/s12978-016-0135-y)
Supplement: Additional file 1: — Peer review reports. (PDF 151 kb) [file 12978_2016_135_MOESM1_ESM.pdf]

Referee's comments to the authors– this sheet WILL be seen by the author(s) and published with the article

Title	**Community perceptions of pre-eclampsia and eclampsia in southern Mozambique**
Author(s)	Helena Boene, Marianne Vidler, Charfudin Sacoor, Abel Nhama, Ariel Nhacolo, Cassimo Bique, Pedro Alonso, Diane Sawchuck, Rahat Qureshi, Eusébio Macete, Clara Menéndez, Peter von Dadelszen, Esperança Sevene, Khátia Munguambe
Referee's name	Keshini Devakandan

When assessing the work, please consider the following points, where applicable:

1. Is the question posed by the authors new and well defined?
2. Are the methods appropriate and well described, and are sufficient details provided to replicate the work?
3. Are the data sound and well controlled?
4. Does the manuscript adhere to the relevant standards for reporting and data deposition?
5. Are the discussion and conclusions well balanced and adequately supported by the data?
6. Do the title and abstract accurately convey what has been found?
7. Is the writing acceptable?

Please make your report as constructive and detailed as possible in your comments so that authors have the opportunity to overcome any serious deficiencies that you find and please also divide your comments into the following categories:

- Major Compulsory Revisions (which the author must respond to before a decision on publication can be reached)
- Minor Essential Revisions (such as missing labels on figures, or the wrong use of a term, which the author can be trusted to correct)
- Discretionary Revisions (which are recommendations for improvement but which the author can choose to ignore)

Where possible please supply references to substantiate your comments.

When referring to the manuscript please provide specific page and paragraph citations where appropriate.

General comments:
As reported in the Methods, 26% of transcripts were translated into English. Was this subset used for the analyses seen in this manuscript? And why was this subset selected instead of a greater number of transcripts?

Major compulsory revisions:

(continue on the next sheet)

Continued:

Minor essential revisions:

The maternal mortality ratio for Sub-Saharan Africa reported in the Abstract is different from that reported in the Background.

The figures are missing labels.

When reporting participant's characteristics in the Results section, marital status is reported for focus group participants, and marital status and gender are reported for interview participants with Table 2 and 3 being referenced, respectively, however these results are not present clearly in the tables.

Some terms in Table 4 are accompanied with the Changana or Portuguese translation. For consistency purposes could all terms be accompanied with a translation, if available, and indication if this translation is in Portuguese or Changana. And the same comments apply to Table 5.

In Table 2, it is not clear why "Ilha-Josina Machel-Calanga" is reported twice. The Methods section indicates that these two posts were combined, thus clarification as to whether these posts were analyzed together or separately is needed. And if these are two separate reports, an alternative name would allow readers to decipher between the two. Also, it is unclear why the "Number of Participants" reported for Women of Reproductive age at Ilha-Josina Machel-Calanga (row 2) is 3(5). This can be explained with a note at the bottom of the table.

Discretionary revisions:

There were a few sentences, which did not flow smoothly. These are found on Line 117, 131 and 285, and specific segments are outlined below.
<u>Line 117:</u> "a Mozambican social scientist (HB), assisted by four local interviewers"
<u>Line 131:</u> "Although the guides were in Portuguese"
<u>Line 285:</u> "The data was <u>collected</u> in four communities"

In the Background, maternal mortality is reported from 1990 to 2013. Is there data from 2000 onwards when the MDGs were adopted? It would be interesting to see how close we are to the 75% reduction rate.

Reviewer 2

Suellen Miller

This paper is very well written, gives appropriate detail for the reader to appreciate the efforts that went into following quality qualitative research methods and has uncovered important information that could be used to change programs that might positively affect behavior and practices. I have suggested some minor changes in "sticky notes" in the text. I believe these changes would enhance the paper. My decision is accept with minor changes, including adding some sentences or a paragraph as to the "how" these findings might be used to improve programs. In the discussion, line 281 the authors mention improving community based health eduction, My comment is: "Perhaps in the discussion section some ways might be suggested based either on other programs in pregnancy in other cultures with similar beliefs or in other medical conditions where perspectives are different between the people experiencing the condition and biomedical perspectives/approaches that might be effective in saving lives?" In the comments I suggest a reference that might be helpful.
